# Supplementary material for: The genomics and evolution of inter-sexual mimicry and female-limited polymorphisms in damselflies
Source: Nat Ecol Evol. 2023 Nov 6;8(1):83–97. doi: 10.1038/s41559-023-02243-1 (PMC10781644; doi:10.1038/s41559-023-02243-1)
Supplement: Supplementary file 2 — Reporting Summary [file 41559_2023_2243_MOESM2_ESM.pdf]

nature portfolio

Corresponding author(s): Beatriz Willink

Last updated by author(s): Sep 19, 2023

Reporting Summary

Nature Portfolio wishes to improve the reproducibility of the work that we publish. This form provides structure for consistency and transparency in reporting. For further information on Nature Portfolio policies, see our Editorial Policies and the Editorial Policy Checklist.

Statistics

For all statistical analyses, confirm that the following items are present in the figure legend, table legend, main text, or Methods section.

n/a	Confirmed	
☐	☒	The exact sample size (*n*) for each experimental group/condition, given as a discrete number and unit of measurement
☒	☐	A statement on whether measurements were taken from distinct samples or whether the same sample was measured repeatedly
☐	☒	The statistical test(s) used AND whether they are one- or two-sided<br>*Only common tests should be described solely by name; describe more complex techniques in the Methods section.*
☒	☐	A description of all covariates tested
☐	☒	A description of any assumptions or corrections, such as tests of normality and adjustment for multiple comparisons
☒	☐	A full description of the statistical parameters including central tendency (e.g. means) or other basic estimates (e.g. regression coefficient) AND variation (e.g. standard deviation) or associated estimates of uncertainty (e.g. confidence intervals)
☒	☐	For null hypothesis testing, the test statistic (e.g. *F*, *t*, *r*) with confidence intervals, effect sizes, degrees of freedom and *P* value noted<br>*Give P values as exact values whenever suitable.*
☒	☐	For Bayesian analysis, information on the choice of priors and Markov chain Monte Carlo settings
☒	☐	For hierarchical and complex designs, identification of the appropriate level for tests and full reporting of outcomes
☒	☐	Estimates of effect sizes (e.g. Cohen's *d*, Pearson's *r*), indicating how they were calculated

Our web collection on statistics for biologists contains articles on many of the points above.

Software and code

Policy information about availability of computer code

Data collection	Software used for base calling in raw ONT data: Guppy v 4.0.11, Guppy v 5.0.11, Guppy v 6.1.5. More details are available in the Methods section.
Data analysis	All code used for data analysis in this study is available at github: https://github.com/bwillink/Morph-locus<br><br>Software used for genome assembly and polishing: Shasta v 0.7.0, minimap2 v 2.22-r1110 83 , PEPPER-Margin-DeepVariant pipeline r0.4, purge_dups v 0.0.3, MaSuRCA v 4.0.4 86, BUSCO v 5.0.0, RagTag v 2.10<br><br>Software used for genome alignment: nucmer v 4.0.0<br><br>Software used for genomic read mapping and pre-processing: bwa-mem v 0.7.17, minimap2 v 2.22-r1110, samtools v 1.9, samtools v 1.14, Picard tools v 2.23.4, GATK v 4.2.0.0<br><br>Software used for variant calling and filtering: bcftools v 1.12, RepeatMasker v 1.0 93, Red v 0.0.1<br><br>Software used for GWAS: PLINK v 1.9<br><br>Software used for k-mer based GWAS: KMC v 3.1.0, GEMMA v 0.98.5, Blast v 2.2.28<br><br>Software used for computing read-depth coverage: mosdepth v 0.2.8

Software used for population genetics analysis: pixy v 1.2.5 (Fst and pi), vcftools v 0.1.17 (Tajima's D)

Software used for calling structural variants: Sniffles v 1.0.10, SamPlot v 1.3.0

Software used for estimating linkage disequilibrium: PLINK v 1.9

Software used for TE annotation: RepeatModeler v 2.0.1, RepeatMasker v 1.0 93, "One code to find them all" v 1.0

Software used for RNAseq read mapping, transcript assembly and count: HISAT2 v 2.2.1, samtools v 1.9, StringTie v 2.1.4

Software used for expression analysis: R v 4.2.2, edgeR v 3.36

Software used for transcript annotation: Transdecoder v 5.5.0, gffread v 0.12.7, Blast v 2.9.0, OrthoFinder v 2.5.2

Software used for PCA: PLINK v 1.9

Software used for plotting: SamPlot v 1.3.0, R v 4.2.2, R packages: RIdeogram v 0.2.2, ggplot2 v 3.4.1, wesanderson v 0.3.6, tidyverse v 1.3.1, dplyr v 1.1.0, gridExtra v 2.3 ggtree v 3.6.2, GenotypePlot v 0.2.1, vcfR v 1.12.0, viridis v 0.6.2, Gviz v 1.38.4

For manuscripts utilizing custom algorithms or software that are central to the research but not yet described in published literature, software must be made available to editors and reviewers. We strongly encourage code deposition in a community repository (e.g. GitHub). See the Nature Portfolio guidelines for submitting code & software for further information.

Data

Policy information about availability of data

All manuscripts must include a data availability statement. This statement should provide the following information, where applicable:

- Accession codes, unique identifiers, or web links for publicly available datasets
- A description of any restrictions on data availability
- For clinical datasets or third party data, please ensure that the statement adheres to our policy

Sequencing data from this study have been submitted to the NCBI Sequence Read Archive (SRA) (https://www.ncbi.nlm.nih.gov/sra/) under accession number PRJNA940276. For individual sample accessions please see Supporting Text 1 and Tables S1 and S2 in the Supplementary Material.

Human research participants

Policy information about studies involving human research participants and Sex and Gender in Research.

Reporting on sex and gender	n/a
Population characteristics	n/a
Recruitment	n/a
Ethics oversight	n/a

Note that full information on the approval of the study protocol must also be provided in the manuscript.

Field-specific reporting

Please select the one below that is the best fit for your research. If you are not sure, read the appropriate sections before making your selection.

☐ Life sciences ☐ Behavioural & social sciences ☒ Ecological, evolutionary & environmental sciences

For a reference copy of the document with all sections, see nature.com/documents/nr-reporting-summary-flat.pdf

Ecological, evolutionary & environmental sciences study design

All studies must disclose on these points even when the disclosure is negative.

Study description

This study consists of several parts with different experimental units. For an overview of analyses and data types see Extended Data Figure 1.

1) De novo genome assembly for female morphs of Ischnura elegans and Ischnura senegalensis. Assemblies were based on one individual sample per morph per species.

2) GWAS, read-depth analysis, population genetics analysis, linkage disequilibrium and SV analysis in Ischnura elegans. These analyses were based on 19 whole-genome resequencing samples of each female morph. See Table S1 for more information on each

	sample. Read-depth analyses and SV calling also used the three long-read sequencing samples used for genome assembly in 1).<br><br>3) Read-depth and SV analysis in Ischnura senegalensis. These analyses were based on two pool-seq DNA samples. Each pool consisted of 30 females of each morph of Ischnura senegalensis.<br><br>4) Expression analysis in Ischnura elegans and Ischnura senegalensis. The analysis in Ischnura elegans was based on 24 RNAseq individual samples, including six A-females, six I-females, six O-females and six males. For each sex/morph category with six samples, three were three sexually immature adults and three were sexually mature adults. This information is also available on Table S2. We also show publicly available data from a previous study on Ischnura senegalensis (Okude et al. PNAS 119, e2114773119). These data correspond to six individuals, two males, two A-females, and two O-females. For each sex/morph category with two individuals, one was sampled upon adult emergence and the other one was sampled two days after emergence. For each of these individuals the head, thorax, abdomen and wings were collected and sequenced separately.
Research sample	Samples were all adult damselflies collected in the field in Southern Sweden (I. elegans), Japan (I. senegalensis, pool-seq data) and Singapore (I. senegalensis, long-read data), as specified in the Methods section. Samples were all female, except for the males used for expression analysis as explained in the "Study description".
Sampling strategy	Individuals were collected upon encounter in the field. There was no statistical analysis to predetermine sample size. The choice of sample size was based on available funding and previous studies with similar types of data.
Data collection	EIS and SN collected all I. elegans samples and recorded their sex, age and morph based on visual inspection. YT and MT collected the pool-seq samples of I. senegalensis and recorded the sex and morph of individuals to be pooled based on visual inspection. BW collected the samples for de novo genome assembly in I. senegalensis and recorded the sex and morph of individuals based on visual inspection.
Timing and spatial scale	I. elegans samples were collected in the summers (June-July) of 2019 (resequencing and RNAseq), and 2020 (genome assembly). I. sengalensis samples were collected in May 2016 (pool-seq) and May and September 2022 (genome assembly). Exact sampling dates for all data except the pool-seq data are available through the Biosample accessions provided in Table S1, Table S2 and the Methods section. Given the goals of this study, the frequency of sampling is not expected to impact any of our results.
Data exclusions	No samples were excluded. SNP data was filtered based on quality and repetitive-content annotations, as explained in the Methods section.
Reproducibility	This study contains no experimental findings.
Randomization	Randomization was not applicable in this study.
Blinding	Blinding was not applicable in this study.

Did the study involve field work? ☒ Yes ☐ No

Field work, collection and transport

Field conditions	Fieldwork consisted only of specimen collection. It was conducted in rural and urban ponds and streams during morning hours on sunny days.
Location	Information on the location of origin of the samples is available through the Biosample accessions on NCBI. See Table S1, Table S2 and the Methods section.
Access & import/export	Collecting permits for insect specimens are only required in Singapore, where research was conducted under a permit from the National Parks Board (NP/RP22-015b). Sequencing was conducted in all cases in the country of origin of the samples, so there was no need for import/export permits. Field sites consisted of ponds and streams in urban or agricultural landscapes. The sites can be easily accessed by researchers and the general public.
Disturbance	The only disturbance caused by the study could have been damage to the surrounding vegetation of ponds and streams as researchers walked and collected specimens. This disturbance is minimal.

Reporting for specific materials, systems and methods

We require information from authors about some types of materials, experimental systems and methods used in many studies. Here, indicate whether each material, system or method listed is relevant to your study. If you are not sure if a list item applies to your research, read the appropriate section before selecting a response.

Materials & experimental systems

n/a	Involved in the study	
☒	☐	Antibodies
☒	☐	Eukaryotic cell lines
☒	☐	Palaeontology and archaeology
☐	☒	Animals and other organisms
☒	☐	Clinical data
☒	☐	Dual use research of concern

Methods

n/a	Involved in the study	
☒	☐	ChIP-seq
☒	☐	Flow cytometry
☒	☐	MRI-based neuroimaging

Animals and other research organisms

Policy information about studies involving animals; ARRIVE guidelines recommended for reporting animal research, and Sex and Gender in Research

Laboratory animals	This study did not involve laboratory animals
Wild animals	Damselfly specimens were collected in the field for sequencing. Individuals were euthanized by freezing or after being submerged in 95% ethanol or liquid nitrogen. Some individuals were euthanized in the field (for RNAseq analysis) while the others were brought to laboratory and immediately preserved.
Reporting on sex	This study addresses the genomics and evolution of a female-limited colour polymorphism, so most individual samples are female. Where data was collected for males also (for RNAseq analysis) the sex of each sample is indicated (See Table S2).
Field-collected samples	Field collected specimens were euthanized in the field or immediately after being brought to a laboratory. Specimens were immediately stored are -20 C or -80 C.
Ethics oversight	No ethical approval or guidance are required for work with insect samples in Sweden, Japan or Singapore.

Note that full information on the approval of the study protocol must also be provided in the manuscript.
